# Methaemoglobinaemia associated with use of glyceryl trinitrate patches in an extremely preterm infant

**DOI:** 10.1111/jpc.15996

**Published:** 2022-04-27

**Authors:** Sujith S. Pereira, Beena Yeldo, Narendra Aladangady

**Affiliations:** ^1^ Neonatal Unit Homerton University Hospital, Homerton Healthcare NHS Foundation Trust London United Kingdom; ^2^ Centre for Genomics and Child Health, Blizard Institute, Barts and the London School of Medicine and Dentistry Queen Mary University of London London United Kingdom

**Key points**

1. GTN patches can cause methaemoglobinaemia and care should be taken to ensure appropriate dosing.
2. Conservative management in this case led to spontaneous resolution of methaemoglobinaemia after removal of GTN patches.
3. Presentation of severe methaemoglobinaemia can be varied and frequent monitoring of methaemoglobin levels is recommended in infants receiving treatment with GTN patches.

Methaemoglobin represents an altered haemoglobin where the iron of heme is oxidised from its normal ferrous (Fe^2+^) form to the abnormal ferric (Fe^3+^) form. Methaemoglobin is unable to bind to oxygen and therefore associated with hypoxia and cyanosis. Normal erythrocytes use nicotinamide adenine dinucleotide (NADH) cytochrome *b*5 reductase to convert the dysfunctional methaemoglobin into functional haemoglobin. Methaemoglobin can occur in newborn infants receiving inhaled nitric oxide therapy and therefore frequent monitoring of methaemoglobin blood levels is undertaken in these infants. Glyceryl trinitrate (GTN), owing to its propensity to cause vasodilatation, has been used to treat ischaemia in adults^1^ and has an unlicensed use for treating ischaemia resulting from complications secondary to arterial catheters or vasopressor extravasation injuries in newborn infants. Though GTN patches are widely used in neonatal intensive care, adverse events have been associated with its use.^2^ We describe in this report, an extremely preterm infant who developed severe methaemoglobinaemia due to inadvertent exposure to high doses of GTN patches which were used to treat tissue ischaemia that occurred following insertion of umbilical arterial catheter (UAC).

## Case Report

A female infant was delivered by Caesarean section at 26 weeks gestation weighing 580 g to a primiparous woman who received two doses of antenatal steroids and magnesium sulphate. Pregnancy was complicated by pre‐eclampsia and intrauterine growth restriction. There was no history of consanguinity or significant maternal medical illnesses. The infant was in good condition at birth with Apgar scores of 9^5^ and 10^10^. The infant developed moderate respiratory distress and was intubated, ventilated and received surfactant soon after birth.

**Fig. 1:**
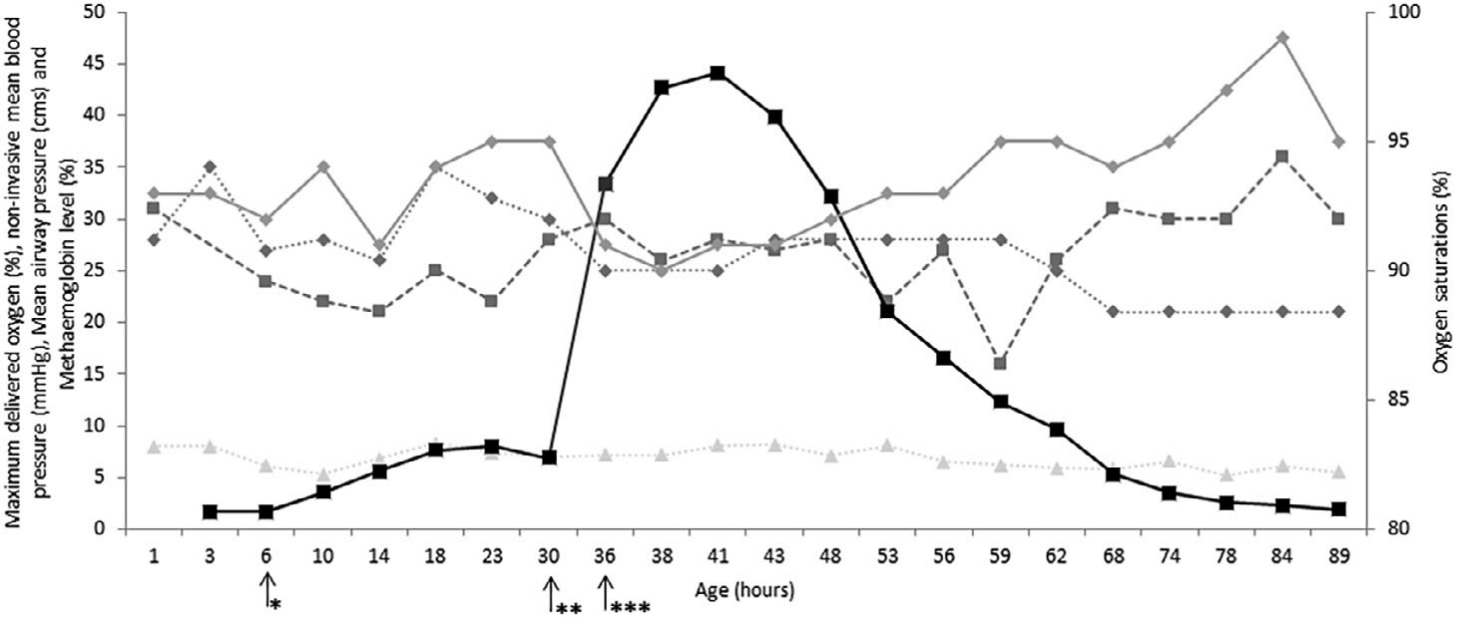
Timeline illustrating the trend in methaemoglobin levels compared with other clinical parameters. Arrows demonstrated when optimum GTN dosage patches were applied (*), inadvertent overdosing occurred (**) and when GTN overdosing ended (***). GTN, glyceryl trinitrate. (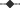) Maximum oxygen delivered (%), (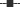) non‐invasive mean BP (mmHg), (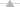) mean airway pressure (cms), (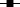) methaemoglobin level (%) and (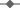) oxygen saturated (%).

In the neonatal unit, umbilical venous catheter (UVC) and UAC were inserted at 2 h of age. After insertion of the UAC, perfusion to the right lower limb was compromised and the limb turned white. The UAC was removed promptly and three 1/4th GTN patches (full patch is 9 cm^2^ patch containing 18.7 mg glyceryl trinitrate) were applied to the affected thigh and leg at 6 h of age. These patches were changed every 24 h. Capillary refill time (CRT) centrally and in the left lower limb was 3–4 s and 5–6 s in the affected right lower limb. The infant received a 0.9% saline bolus (10 ml/kg) to increase circulating volume. At 31 h of age, when the GTN patches were changed, three full GTN patches were inadvertently applied to the affected limb for a period of 5 h resulting in a steep rise in methaemoglobin levels as shown in Figure 1. There was a corresponding reduction in oxygen saturation levels. There was no associated drop in blood pressure. All GTN patches were promptly removed and frequent monitoring of methaemoglobin levels demonstrated a significant reduction to normal levels over the next 48 h. Perfusion to the limb improved (CRT of 3–4 s) and the limb appeared dusky rather than white by 40 h of age. The infant remained ventilated on minimal respiratory support. Ultrasound and Doppler investigations of the abdominal aorta and femoral artery showed no evidence of thrombus. However, the size of the right femoral artery was smaller compared to the left femoral artery. Echocardiography showed a 2 mm patent ductus arteriosus with good cardiac filling and optimal cardiac output. Serial cranial ultrasound scans were normal. The perfusion to the right leg was fully restored with no loss of tissue and the infant made a full recovery by day 4. The infant was establishing feeds and was extubated to non‐invasive respiratory support by day 5. The infant continued to progress well with minimal oxygen and normal methaemoglobin levels.

## Discussion

This case illustrates acquired methaemoglobinaemia in an extremely preterm infant resulting from inadvertent overdosing with GTN patches which were used to treat limb ischaemia, a well‐documented complication of UACs. The absence of consanguinity, lack of cyanosis soon after birth and normal methaemoglobin level at birth make congenital methaemoglobinaemia less likely. Reduction and normalisation in methaemoglobin levels after removing the GTN patches makes overdosing the likely aetiology for methaemoglobinaemia.

Methaemoglobinaemia is a well‐documented complication of inhaled nitric oxide use in newborn infants.^3^ GTN patches are commonly used as first‐line agents in arterial catheter induced ischaemia.^4^ GTN is converted to nitric oxide and activates guanylate cyclase thus increasing levels of cyclic guanosine monophosphate (cGMP) which causes vasodilatation and improved circulation.^5^ Methaemoglobinaemia has been reported in two extremely preterm infants (maximum level of 8.4% and 23.2% in infants born at 24 weeks gestation) following GTN patch application.^6^ These infants had significant rise in oxygen requirements with one infant developing necrosis of the digit. In our case, there was a slight increase in methaemoglobin (5–6%) following the initial use of optimum dose of GTN patches, and reached a maximum methaemoglobin level of 44.1% following inadvertent exposure to high dose of GTN patches. This was associated with a drop in peripheral oxygen saturation levels, but maximum oxygen requirement at this point was only 25%. Systematic review of topical nitroglycerin ointment as salvage treatment for tissue ischaemia in newborns concluded that topical nitroglycerin ointment had a favourable efficacy‐safety profile in preventing morbidities when compared with GTN patches and sprays.^2^

Dosing for GTN can be variable with other modes of medication delivery such as ointments and sprays. GTN patches also have less flexibility in dosing thus potentially increasing the risk of adverse outcomes (overdosing) or therapeutic failure (underdosing). There is limited data for recommending GTN sprays and the recently published systematic review favoured topical nitroglycerin ointment.^2^ Premature infants are more prone to methaemoglobinaemia than infants born at term due to multiple reasons such as skin immaturity causing increased absorption of the medication, lower levels of NADH cytochrome *b*5 reductase levels^7^ and higher levels of fetal haemoglobin which can easily convert to methaemoglobin.^8^

Methaemoglobinaemia could be potentially life‐threatening in extremely preterm infants. We recommend regular monitoring of methaemoglobin levels whilst infants are treated with GTN patches for ischaemia, and to act appropriately on noticing increasing methaemoglobin to prevent serious complications of methaemoglobinaemia.

## References

[1] Boyce L , Dhukaram V . Transdermal glyceryl trinitrate in the treatment of ischemia following toe deformity correction: A case series. Foot Ankle Int. 2014; 35: 1226–30. 25125514 10.1177/1071100714546887

[2] Sushko K , Litalien C , Ferruccio L *et al*. Topical nitroglycerin ointment as salvage therapy for peripheral tissue ischemia in newborns: A systematic review. CMAJ Open 2021; 9: E252–E60. 10.9778/cmajo.20200129 PMC8096410 33731426

[3] Hamon I , Gauthier‐Moulinier H , Grelet‐Dessioux E , Storme L , Fresson J , Hascoet JM . Methaemoglobinaemia risk factors with inhaled nitric oxide therapy in newborn infants. Acta Paediatr. 2010; 99: 1467–73. 20456277 10.1111/j.1651-2227.2010.01854.x

[4] Adiotomre P , Elliot L . Yorkshire & Humber Neonatal Network (South) Clinical Guideline: Extravasation Injuries in Neonates. Yorkshire and Humber Neonatal Operational Delivery Network. 2018. Available from: https://www.networks.nhs.uk/nhs-networks/yorkshire-humber-neonatal-odn/guidelines-1/guidelines-new/skin/extravasation-injuries

[5] Ignarro LJ , Cirino G , Casini A , Napoli C . Nitric oxide as a signaling molecule in the vascular system: An overview. J. Cardiovasc. Pharmacol. 1999; 34: 879–86. 10598133 10.1097/00005344-199912000-00016

[6] Mintoft A , Williams E , Harris C , Kennea N , Greenough A . Methemoglobinemia during the use of glyceryl trinitrate patches in neonates: Two case reports. AJP Rep. 2018; 8: e227–e9. 30345159 10.1055/s-0038-1669945 PMC6188887

[7] Kravitz H , Elegant LD , Kaiser E , Kagan BM . Methemoglobin values in premature and mature infants and children. AMA J. Dis. Child. 1956; 91: 1–5. 13275111 10.1001/archpedi.1956.02060020003001

[8] Johnson SF . Methemoglobinemia: Infants at risk. Curr. Probl. Pediatr. Adolesc. Health Care 2019; 49: 57–67. 30956100 10.1016/j.cppeds.2019.03.002

